# Reduced resting-state brain activity in the default mode network in children with (central) auditory processing disorders

**DOI:** 10.1186/1744-9081-10-33

**Published:** 2014-09-26

**Authors:** Agnieszka Pluta, Tomasz Wolak, Natalia Czajka, Monika Lewandowska, Katarzyna Cieśla, Mateusz Rusiniak, Diana Grudzień, Henryk Skarżyński

**Affiliations:** 1 World Hearing Center of the Institute of Physiology and Pathology of Hearing, Mokra 17 street, 05-830 Nadarzyn, Warsaw/Kajetany, Poland

**Keywords:** Default mode network, Independent component analysis, Regional homogeneity, Functional magnetic resonance imaging, Central auditory processing disorders

## Abstract

**Background:**

In recent years, there has been a growing interest in Central Auditory Processing Disorder (C)APD. However, the neural correlates of (C)APD are poorly understood. Previous neuroimaging experiments have shown changes in the intrinsic activity of the brain in various cognitive deficits and brain disorders. The present study investigated the spontaneous brain activity in (C)APD subjects with resting-state fMRI (rs-fMRI).

**Methods:**

Thirteen children diagnosed with (C)APD and fifteen age and gender-matched controls participated in a rs-fMRI study during which they were asked to relax keeping their eyes open. Two different techniques of the rs-fMRI data analysis were used: Regional Homogeneity (ReHo) and Independent Component Analysis (ICA), which approach is rare.

**Results:**

Both methods of data analysis showed comparable results in the pattern of DMN activity within groups. Additionally, ReHo analysis revealed increased co-activation of the superior frontal gyrus, the posterior cingulate cortex/the precuneus in controls, compared to the (C)APD group. ICA yielded inconsistent results across groups.

**Conclusions:**

Our ReHo results suggest that (C)APD children seem to present reduced regional homogeneity in brain regions considered a part of the default mode network (DMN). These findings might contribute to a better understanding of neural mechanisms of (C)APD.

## Background

Some children with school difficulties report increased susceptibility to distraction, trouble understanding speech in the presence of a competing background noise, following rapid speech and talking on the phone. These might be manifestations of central auditory processing disorder (C)APD, involving deficits in one or more of the following functions: sound localization and lateralization, auditory discrimination and pattern recognition, temporal aspects of audition and performance with competing and degraded acoustic signals [1]. (C)APD is diagnosed based on behavioral tests after normal peripheral hearing had been confirmed and originates from failure to process, comprehend or/and respond efficiently to the incoming auditory stimuli [2,3].

(C)APD can result from different etiologies but a substantial number of children diagnosed have diffuse dysfunction of the central nervous system (CNS) with no identifiable lesions. Among factors which may underlie the wide range of listening problems, researchers consider delayed CNS maturation and neurological conditions [2,4]. Therefore, brain imaging methods (such as fMRI, PET, EEG) may extend knowledge about neural mechanisms underlying (C)APD and, thus, improve the diagnostics [5]. Nevertheless, up to date, there have only been several such studies published [4,5]. A case study of a patient with (C)APD [4], combining MRI and PET, revealed structural and functional changes (encompassing atrophy and high signal intensity in bilateral auditory cortices, hypometabolism in the primary and the secondary auditory cortex and precuneus, as well as hypermetabolism in caudate and superior frontal sulci). However, the patient additionally demonstrated severe problems with speech understanding and production and slightly degraded hearing acuity, and therefore these findings cannot be generalised over the whole population with (C)APD.

Recently, there is a growing interest in the examination of temporal correlations between various brain regions during unconstrained intrinsic activity (rs-fMRI), as researchers claim that specific alterations might be implicated in various cognitive deficits and brain disorders [6-8].

One of the most widely examined intrinsic networks is the default mode network (DMN). The term ‘DMN’ describes a set of functionally and anatomically organized regions that show increased synchronization during rest and are suppressed during task performance. DMN encompasses the precuneus/posterior cingulate (pCu/PCC), the areas of frontal pole (eg. BA 10) and posterior parietal cortices [9]. DMN is considered to be involved in internally generated tasks, such as attending to environmental stimuli or anticipating upcoming events [9]. The interaction of DMN regions and brain areas involved in cognitive control is considered to provide balance between internally and externally directed thoughts and thus may be implicated in the regulation of focus of attention [10]. In line, the newest research demonstrates that human vigilance can be directly related to the activity of the default-mode network [11] and an atypical pattern in DMN can be associated with attention impairments [12,13]. This issue is not well understood but many studies have linked changes in DMN with attentional problems (see [14] for a review).

Since a close, predictable relationship between auditory processing performance and attention was identified [1], DMN seems to be a relevant measure of brain activity in (C)APD. Although there is limited data available concerning neural underpinnings of (C)APD specifically, extensive research has been conducted in patients without (C)APD diagnosis who nevertheless demonstrate auditory processing problems, such as in ADHD [15,16]. Due to the fact that (C)APD and ADHD frequently co-occur and share some behavioural symptoms, particularly inattention and distractibility [17], some authors claim that overlapping cognitive mechanisms might account for both diseases. The common manifestations may, therefore, result from aberrations in distributed brain networks with associated central auditory pathways [3].

For instance, Castellanos and colleagues observed decreased functional connectivity between the mPFC (medial prefrontal cortex) (BA 9 and BA 10) and PCC regions in children with ADHD [18], whereas Cao and collaborators [19] demonstrated decreased regional homogeneity in the frontal-striatal-cerebellar circuits in this group of patients (with auditory processing difficulties). Interestingly, many authors argue that disintegration (e.g. attenuated coherence or less efficient deactivation) of DMN regions may cause interference during focus of attention [20,21].

In summary, central auditory processing difficulties demonstrated in various groups of patients have been associated with structural and functional brain abnormalities but the exact neural correlates of (C)APD need further thorough investigation with the use of modern non-invasive neuroimaging techniques.

Although, resting state-fMRI studies have not been performed previously in individuals with (C)APD, lapses of attention observed in other developmental attention disorders were considered to be associated with alternations in DMN. Therefore, we were motivated to investigate whether there are DMN aberrations specific for children with (C)APD without coexisting ADHD, language and emotional problems. Based on some other studies in subjects with attention problems, we hypothesized that children with auditory processing difficulties would have abnormal (decreased or increased) synchronization in brain regions corresponding to DMN. In this study, we focused on DMN because brain regions within this network are considered to subserve attentional modulation that is impaired in (C)APD. According to the authors’ knowledge, this is the first fMRI study to apply both ReHo and ICA methods for data analysis to investigate spontaneous brain activity within children with (C)APD.

## Methods

### Subjects

The experimental group consisted of 13 children with (C)APD (7 girls, 6 boys, aged: 7.3-16.0 years, mean = 12.2) and 15 control children (7 girls, 8 boys, aged: 7.0-16.0 years, mean = 11.7). There was a comparable number of girls and boys in each group and the groups were not significantly different with respect to chronological age (t = -0.5; p > 0.05).

Children with (C)APD were recruited from the patients of the Institute of Physiology and Pathology of Hearing, prior to their participation in a specifically-designed therapeutic programme serving to improve auditory processing and attention. The control group was recruited *via* an advertisement in local press. Control subjects obtained results within age-specific reference ranges in a (C)APD examination according to the Polish norms and did not have any history of neurodevelopmental disorders and/or learning problems.

(C)APD diagnosis was made by a multidisciplinary team comprising audiologists, speech-language therapists, psychologists, educators, and physicians. Children who had ADHD symptoms, language problems, psychiatric disorders and/or emotional disturbances were not included in the study.

The (C)APD was diagnosed based on the performance in the following tests: Frequency Pattern Test (FPT), Duration Pattern Test (DPT), Gap Detection Test (GDT), Dichotic Digit Test (DDT) and Adaptive Speech in Noise Test (ASpN), as recommended for (C)APD screening [1]. Each child diagnosed with (C)APD displayed abnormal performance in all tests, compared with reference results of Polish native speaking children. Detailed behavioural findings in both study groups are provided in Table 1 in the Results Section.

**Table 1:** Clinical characteristics

**Test**	**(C)APD group**	**Control group**	**Two-sample t-test**
	**X (SD)**	**X (SD)**	
FPT	44.4 (17.5)	81.66 (11.6)	6.6**
DPT	57.8 (21.7)	87.2 (11)	4.4**
DDT right ear	74.2 (13.6)	87.5 (11)	2.8**
DDT left ear	64.8 (19.3)	80.5 (15.3)	2.4*
GDT	3.7 (1.3)	2.3 (2)	-2.2*
aSPN	0 (2.3)	-1.9 (1.3)	-2.6*

*p < 0.05, **p < 0.01; *Abbreviations*: *SD* standard deviation; *FPT* Frequency Pattern Test; *DPT* Duration Pattern Test; *GDT* Gap Detection Test; *aSPN* adaptive Speech In Noise. If not otherwise indicated, the stimulation was presented binaurally. We measured: the percentage of correct responses in FPT, DPT and DDT; the minimal detected gap duration (in milliseconds) in GDT; the minimal SNR for 50% correct word recognition in aSPN.

All children participating in the study were right-handed, had normal hearing (as measured with tonal audiometry at the Institute of Physiology and Pathology of Hearing) and intellectual abilities within the normal range (Raven’s Progressive Matrices). They also had no history of neuropsychiatric disorders, head trauma or medication affecting the CNS. Subjects’ parents provided both oral and written informed consent in compliance with the Code of Ethics of the World Medical Association (Declaration of Helsinki) and guidelines of the Bioethics Committee of the Institute of Physiology and Pathology of Hearing. Participants were informed that they can withdraw from the study at any moment.

### Imaging protocol

A 3 T Siemens Magnetom Trio Tim MR scanner equipped with a 12-channel matrix head coil and MR-compatible goggles (Nordic NeuroLab Visual system) were used to perform the imaging study. The scanning sessions included: 1) localization; 2) 3D T1-weighted image acquisition covering the entire brain (3D MP-RAGE sequence, TR = 1900 ms, TE = 2.26 ms, 0.9 × 0.9 × 0.9 mm voxels); 3) resting-state functional imaging (TR = 2000 ms, TE = 30 ms, flip angle = 90°, FOV 192 mm, matrix size 64 × 64, scanned volume-37 axial sequential slices of 3.5 mm thickness [no gap, 3 × 3 × 3.5 mm voxel]), AC-PC oriented, 150 volumes, iPAT = 2). The resting-state protocol was 5 minutes in duration, producing 150 brain volumes. During the study, the subjects were instructed to remain motionless, keep their eyes open (the subjects were presented with a black screen) and try to relax.

### ReHo and ICA analysis

The first 10 volumes were discarded. The processing steps were the same for ReHo and ICA: 1) slice timing (correction to the first slice), 2) realignment (a detailed description is presented below in section Motion and data quality analysis), 3) normalization to the MNI template, 4) detrending, 5) for ReHo: temporal band–pass frequency filtering (0.01-0.08 Hz) and for ICA: spatial smoothing with a Gaussian kernel of 6 mm full width at half maximum (FWHM). The preprocessing was performed in DPARSF (http://rfmri.org/DPARSF).

Once pre-processing was completed, REST software (http://resting-fmri.sourceforge.net) was used to obtain ReHo maps (by calculating Kendall’s concordance coefficient value to measure the similarity of the ranked time series of a given voxel to its nearest 27 neighbor voxels in a voxel-wise way). After obtaining individual ReHo maps the data were smoothed with a Gaussian kernel (FWHM = 4 mm). First, one-sample t-tests were performed for the inter-subject analysis of each voxel of each image to identify voxels that had significantly higher ReHo. Then, a two-sample t-test was computed on the normalized individual ReHo maps to investigate ReHo differences between the two groups.

The Matlab toolbox, group ICA of fMRI Toolbox (GIFT; http://icatb.sourceforge.net), was used for ICA analysis. This included: principal component analysis (PCA) reduction, ICA separation and back-reconstruction to produce single-subject time courses and spatial maps from the raw data matrix [22].

The dataset was decomposed with different model orders: 29 (the optimal number of group’s components based on minimum description length criteria for source estimation); 20 (a default number of components in GIFT); 40 and 50 components. The rationale for using ICA with different numbers of independent components was that according to other studies, lower model orders provide a general insight into large-scale networks, whereas higher model orders can serve to detect more fine-grained networks corresponding to anatomical and functional segmentations [23,24]. The data were separated by ICA using Infomax algorithm. For each model order, the ICA approach was repeated 30 times with ICASSO implemented in the GIFT toolbox.

Group ICA was run with all subjects included in one group, to ensure that the same components were identified in each subject. The default mode component was identified by spatially correlating all components with two default mode masks provided in GIFT: “DMN_ICA_REST.Nii” and “ref_default_mode.nii”. These masks contained regions that have been most commonly reported to comprise the DMN [25,26]. We used different templates as they seem to be more weighted on different nodes of DMN: ref_default_mode.nii enables to sort the more posterior part of DMN, whereas DMN_ICA_REST sorts more anterior hubs. The component that most significantly (spatially) correlated with each template were selected as the default mode network. Then, the DMN components were transformed to z-scores using the GIFT software and exported to SPM8 (Wellcome Trust Centre for Neuroimaging, http://www.fil.ion.ucl.ac.uk/spm) where statistical analysis was performed. Statistical maps of the default mode network for patients and comparison subjects were created by entering the single-subject default mode component into a voxelwise one-sample t test and subsequently a two-sample t test, which was then thresholded at p < 0.05 with AlphaSim Correction. Regions were labelled using the Talairach Daemon Atlas [27].

### Motion and data quality analysis

In order to eliminate the possible influence of motion on the data we followed the procedures described by van Dijk and collaborators [28]. First, separate matrices encompassing mean motion, maximum motion, and rotation of head motion were calculated based on translation and rotation parameters [28,29]. Subjects whose mean motion parameters exceeded 0.5 mm were discarded from further analysis. Therefore, the final (C)APD study group consisted of 13 out of 23 recruited children with (C)APD and 15 out of 30 children were selected from among the examined healthy controls.

To make sure that the final (C)APD and healthy controls groups had similar head motion characteristics, we evaluated the maximum between-group head motion differences. The group mean results and group differences (two-sample t-test) were as follows:

– mean motion of 0.1 (SD = 0.09) mm for (C)APD and 0.12 (SD = 0.1) for healthy controls (p = 0.405);

– maximum motion of 1.1 (SD = 1.2) mm for (C)APD and 1.1 (SD = 0.9) for healthy controls (p = 0.99);

– mean rotation of 0.055 (SD = 0.06) mm for (C)APD and 1.1 (SD = 0.65) for healthy controls (p = 0.703).

Secondly, the temporal signal-to-noise ratio (tSNR) parameter was estimated to evaluate the quality of the data and to eliminate any possible causes of data instability [28]. The mean tSNR for the (C)APD group was 289 (SD = 106), and the mean tSNR for healthy controls was 253.1 (SD = 113). A two-sample t-test showed no between-group differences [t (26) = 1.036, *p* = 0.310].

## Results

### Clinical characteristics

The (C)APD group performed significantly poorer than controls in all tests that were applied (see: Table 1).

### Resting-state brain activity patterns in (C)APD and in the control group

With the use of ReHo analysis in both groups we found a pattern of brain regions consistent with the DMN nodes provided in literature [9,13,26]. Nevertheless, two-sample t-test showed inter-group differences in regions encompassing: the precuneus (pCu)/posterior cingulate cortex (PCC) and the area of frontal pole corresponding to BA 10.

**Figure 1:**
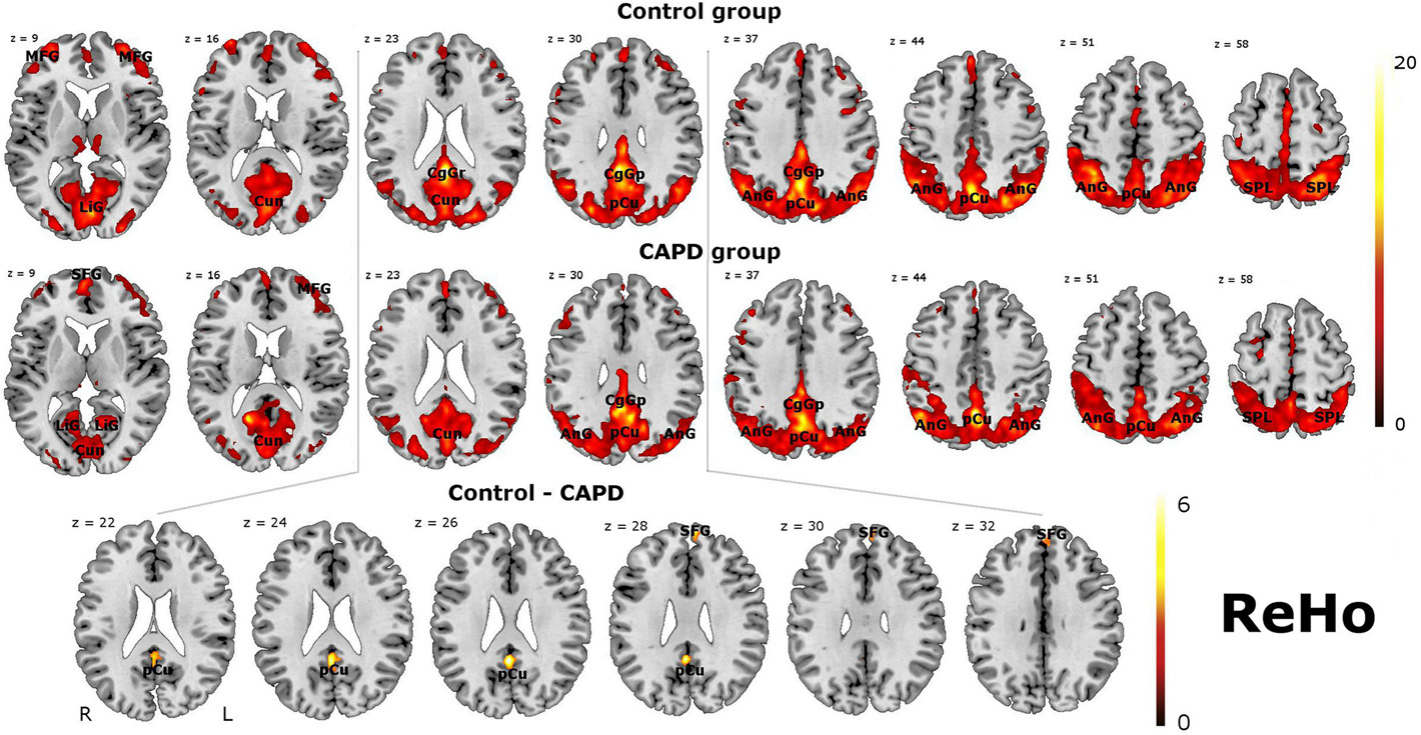
**Cortical representations of DMN obtained with the ReHo method in (C)APD and the control subjects and a comparison between normal controls and patients with (C)APD (maps thresholded at *p*** **< .005, AlphaSim corrected, no of voxels > 66).** The color scale represents *T* values.

Table 2 provides a list of brain regions in DMN, along with coordinates of peak voxels and the number of activated voxels obtained in ReHo. Results obtained with the use of ReHo are presented in Figure 1. The final statistical map was set at a combined threshold of P < 0.005 and a minimum cluster size of 66 voxels, which resulted in a corrected threshold of P < 0.05 determined by AlphaSim.

**Table 2:** Clusters showing significant group differences in ReHo (Controls > (C)APD)

**Brain area**	**Cluster coordinates**			**Voxel peak T**	**Number of activated voxels**
	**x**	**y**	**z**		
pCu/PCC	2	-50	24	5.57	86
Superior Frontal Gyrus	-4	58	28	4.30	51

*Abbreviations*: *pCu* precuneus; *PCC* posterior cingulate cortex.

**Figure 2:**
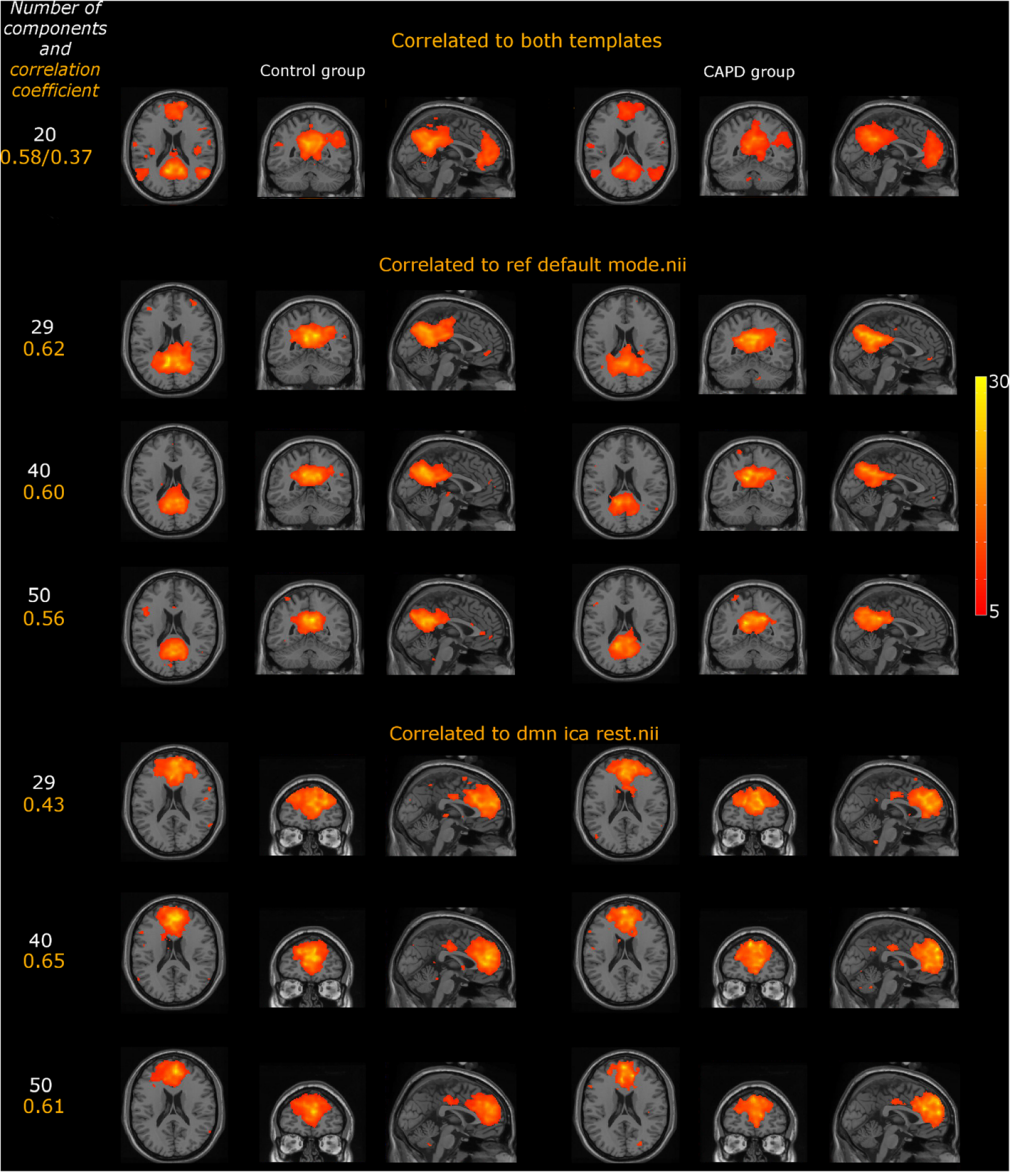
**Cortical representations of DMN obtained with the ICA method for different model settings in (C)APD, and the control subjects (maps thresholded at p < .005, AlphaSim corrected, no of voxels > 66).** The color scale represents *T* values.

For ICA, the topographic distribution of DMN was similar in both groups and encompassed: middle frontal gyrus, posterior cingulate, precuneus, lateral parietal lobule, bilaterally. For the model with 20 components we found one component corresponding to DMN, regardless of the chosen template (correlation values for ref_default_mode.nii and DMN_ICA_REST. nii were 0.58 and 0.37, respectively). Nevertheless, we found that for model orders 29, 40, 50 the areas of significant co-activation start to decompose into the posterior and the anterior DMN. Interestingly, for the two DMN templates two different patterns of co-activation consistent with DMN (correlation values ranging from 0.43 to 0.65) were found. Consistently, depending on the model order and the components identified based on the correlation coefficient with the two templates inter-group results were inconsistent. Therefore, for the sake of clarity, we decided to present within-group results only (one-sample t-test). The final statistical map was set at the same threshold as in case of ReHo.Results obtained with the use of ICA are shown in Figure 2. In addition Figure 2 provides the correlation values for DMN identified based on the two described templates.

## Discussion

In this paper we examined resting-state brain activity in children with (C)APD, using two independent data-driven approaches: ReHo and ICA with different model order settings.

First, we should emphasize that to date there has only been one preliminary study published which aimed to develop an fMRI procedure to examine individuals with (C)APD [5]. According to the authors’ best knowledge, this is the first study which applied rs-fMRI to investigate brain activity in (C)APD. Secondly, we used two different methods (ReHo and ICA) to analyze the same data set, in order to provide a more complex picture of DMN.

On the basis of one-sample *t*-tests performed in children with (C)APD and in the control group, with the use of two analysis methods, we clearly demonstrated patterns of activity consistent with the default mode network [26]. However, ReHo analysis revealed differences in resting-state brain activity in the (C)APD group, relative to controls. Specifically, a significant ReHo decrease was found in the PCC/pCu and the SFG (BA 10) in patients. The ICA showed that the model order selection, as well as component selection can both significantly affect the results. Therefore, due to inconsistent results in two-sample t-tests, drawing conclusions about group differences based on ICA does not seem eligible.

### Rs-fMRI in (C)APD

In the present study we used a rs-fMRI paradigm to investigate brain activity in (C)APD (and not an active task). There were several reasons why we did so. First, previous fMRI studies in children with (C)APD showed that their concentration and tests performance decreases with increased duration of examination [5]. Then the possible group differences in brain activations may results from attentional lapses, only caused by the duration of the experiment rather than the modality-specific impairment. Resting state-fMRI, which is of very short duration, enables to overcome this limitation. Secondly, a paradigm that does not require children to cooperate actively, might diminish motion artifacts, resulting from reacting to stimulation.

Furthermore, a rs-fMRI paradigm allows us to examine a whole brain network rather than focal activations associated with a particular task. As (C)APD children in the present study showed deficits in all behavioral (C)APD screening tests, it would be advisable to apply as many as five different cognitive tasks in an MR scanner in each child. It would not be possible within a single fMRI session and it would be problematic to ask the subject to participate in an fMRI experiment several times in a short period of time.

### Default mode network and attention

The ReHo results of the study are interesting, as the posterior cingulate cortex (PCC) and the precuneus not only form a central node in DMN in the human brain but they have also been suggested to play an important role in attention and goal-directed cognition [30,31]. PCC shows high connectivity to the frontal network involved in cognitive control and attention demanding tasks [30]. Moreover, a specific pattern of PCC/pCu activity has been found in several rs-fMRI studies in ADHD [18].

Additionally, altered connectivity between the anterior (prefrontal cortex) and the posterior (posterior cingulate/precuneus) components of the DM network [18,32] has been reported in subjects with ADHD. In our study we did not assess the functional connectivity directly, but we also found alternations in corresponding regions in ReHo analysis. As ReHo corresponds to connectivity at the local level, the revealed decreased ReHo values may reflect low regional metabolism [33] in PCC/pCu and the SFG (BA 10). This is in line with other studies linking attention disorders with disrupted consolidation of the DMN [18].

The atypical activity found in the region of PCC may suggest similar brain mechanisms underpinning (C)APD and ADHD. This can also indicate that rs-fMRI cannot differentiate between the modality-specific attention problems observed in (C)APD and general attention impairments.

A number of studies have suggested that dysregulation of DMN may be associated with lapses of attention and errors during cognitively engaging tasks [14]. According to default-mode interference hypothesis inefficacious transition from rest to task may account for impaired cognitive task performance [13]. It deserves further consideration, to investigate whether errors observed in (C)APD diagnostic batteries originate from general attention problems or a modality-specific perceptual dysfunction, as suggested by several authors [34]. Interpretative issues related to ICA with regard to the DMN analysis are discussed in the next section.

### ICA or ReHo?

In the present study two different methods (ICA and ReHo) to analyze the rs-fMRI data were applied. Except for purely methodological studies, researchers usually choose only one approach to analyze the data. This may lead to some discrepancies between studies using different analysis techniques [35] or even only ICA but with different model settings [23].

One of the most popular techniques applied in resting-state fMRI studies has been ICA [36]. The main advantage of this method is lack of any initial assumptions about the spatial location of brain activations, which makes it suitable for exploratory fMRI analysis [36]. ICA is, however, not without its challenges, as it requires an arbitrary decision on the dimensionality reduction, as the number of components to estimate is not fixed and should be selected according to the data quality and the complexity of the results one wishes to obtain [e.g. resting state networks (RSN) may be separated into sub-networks reflecting their fine-grained nature] [36,37]. Also, components representing networks of interest might be selected differently based on various templates which may lead to divergent results, as was presented in our study. Therefore, the reproducibility of the results obtained with ICA might be questioned, which has also been emphasized by other authors [23]. The issue of inconsistent ICA results has hardly been discussed and not resolved yet [23]. Thus, group ICA should be used with caution when drawing inferences about group differences.

Another approach widely used to the evaluation of rs-fMRI is regional homogeneity (ReHo), which investigates the temporal congruency of the regional BOLD signal in various brain regions [38,39]. Both ReHo and ICA represent a connectivity analysis but they are performed with different mathematical approaches. While ReHo reveals correlations between slow fluctuations of the BOLD MR signal in various brain regions, ICA determines networks related to the decomposed temporal signal fluctuations. Importantly, ReHo is relatively insensitive to possible region-to region or trial-to-trial variability of the BOLD signal, which makes it complimentary to ICA [36]. The possible discrepancies in the results (which were demonstrated in our study) may be due to the fact that in ICA, the mean time course of a whole network is compared with the time course of individual voxels within that network [40], whereas ReHo seeks for correlations between the BOLD fluctuations of a small group of voxels with the adjacent ones. This might suggest that ReHo is more sensitive to local changes of functional connectivity and the ICA approach examines long-range connectivity [13].

Additionally, in ICA, due to differences in the model settings used in various studies results might be not reproducible. Also, possible inter-group differences might be concealed, as they may emerge in the components not included in further analysis.

Taking into account the aforementioned considerations, we recommend ReHo as the method providing more stable results in DMN network compared to the ICA.

### The effect of motion

As a number of studies indicate that disruptions in BOLD signal resulting from head motion may create spurious patterns of correlations in rs-fMRI we made sure that in our study the two groups did not differ either in their motion parameters or in tSNR [29,41]. Originally our study group was twice as big but after the motion parameter analysis we decided to eliminate participants whose motion might have confounded the results. We decided to exclude participants rather than apply “scrubbing” methods, as it was reported that this procedure does not always remove all motion-related signal and that these are most efficient when periods of motion are within a single TR [41]. In children, however, the periods of motion span across multiple TRs. Consequently, replacing time points in which movements occurred with adjacent ones may contribute to losing the signal of interest.

### Limitations of the study

The presented findings are promising but several methodological limitations should be indicated (which are not confined to the present study). First, although there is a growing interest in rs-fMRI studies, interpretative issues remain to be resolved before the alternations in the intrinsic brain activity can serve as an indicator of cognitive dysfunction [36].

We suggest that group differences, showed in the ReHo analysis, reflect limited regional neuronal synchronization in (C)APD, which might mean that detected neurons do not behave in a coherent fashion during rest. However, considering the fact that physiology underlying spontaneous BOLD response might be different to that when performing a task, it is under debate whether alternations found in this study directly underlie behavioral problems manifested by individuals with (C)APD.

Finally, although we performed ICA with different model orders, we only selected components which represented DMN and compared that particular components with the results obtained with ReHo. The rationale was that from all the reported resting-state brain networks, only DMN is considered to be involved in the introspective modes of cognition, encompassing the shift between the internally and the externally directed attention [6]. Nevertheless, it is possible that selecting components reflecting different resting state networks would provide additional valuable results.

Since the ReHo approach revealed significant differences in DMN in children with (C)APD, relative to controls, we conclude that this matter deserves further consideration. Applying different neuropsychological and neuroimaging methods (including also task-based fMRI paradigms) might be helpful in differentiating developmental CAPD from other developmental cognitive disorders and further elucidate the neural basis of (C)APD.

## Conclusions

In conclusion, our study demonstrates that the ReHo approach is sensitive to detect alternations in spontaneous brain activity in children with (C)APD, and thus might potentially help elucidate neuro-cognitive mechanisms underlying behavioral problems manifested in this patient population. Nevertheless, both ReHo and ICA methods should be used with caution especially when inferences about group differences are drawn.

## Abbreviations

AnG: Angular gyrus; pCu: Precuneus; CgGr: Cingulate gyrus, rostral (anterior) part; CgGp: Cingulate gyrus posterior part; SFG: Superior frontal gyrus; SPL: Supraparietal lobule; MFG: Middle frontal gyrus; Cun: Cuneus; LiG: Lingular gyrus; PCC: Posterior cingulate cortex; PCg.

## Competing interests

The authors do not have an affiliation with or financial interest in any organization that might pose a competing interest.

## Authors’ contributions

AP and TW took part in designing the research, data analysis and the manuscript preparation. NC and DG collected and analyzed the behavioral data. ML and KC took part in the manuscript preparation. MR took part in data analysis and interpretation. HS provided valuable comments and remarks on the manuscript and supervised the interpretation of the results. All authors read and approved the final manuscript.

## References

[B1] Moore DR Ferguson MA Edmondson-Jones AM Ratib S Riley A Nature of auditory processing disorder in children Pediatrics 2010 126 e382 e390 10.1542/peds.2009-2826 20660546

[B2] Bamiou D-E Musiek FE Luxon LM Aetiology and clinical presentations of auditory processing disorders—a review Arch Dis Child 2001 85 361 365 10.1136/adc.85.5.361 11668093 PMC1718975

[B3] Bailey T Auditory pathways and processes: implications for neuropsychological assessment and diagnosis of children and adolescents Child Neuropsychol 2010 16 521 548 10.1080/09297041003783310 20924853

[B4] Kim M-J Jeon H-A Lee K-M Son Y-D Kim Y-B Cho Z-H Neuroimaging features in a case of developmental central auditory processing disorder J Neurol Sci 2009 277 176 180 10.1016/j.jns.2008.10.020 19058816

[B5] Bartel-Friedrich S Broecker Y Knoergen M Koesling S Development of fMRI tests for children with central auditory processing disorders In Vivo 2010 24 201 209 20363995

[B6] Snyder AZ Raichle ME A brief history of the resting state: the Washington University perspective NeuroImage 2012 62 902 910 10.1016/j.neuroimage.2012.01.044 22266172 PMC3342417

[B7] Assaf M Jagannathan K Calhoun VD Miller L Stevens MC Sahl R O’Boyle JG Schultz RT Pearlson GD Abnormal functional connectivity of default mode sub-networks in autism spectrum disorder patients NeuroImage 2010 53 247 256 10.1016/j.neuroimage.2010.05.067 20621638 PMC3058935

[B8] Camchong J MacDonald AW Bell C Mueller BA Lim KO Altered functional and anatomical connectivity in schizophrenia Schizophr Bull 2011 37 640 650 10.1093/schbul/sbp131 19920062 PMC3080691

[B9] Buckner RL Andrews-Hanna JR Schacter DL The brain’s default network: anatomy, function, and relevance to disease Ann N Y Acad Sci 2008 1124 1 38 10.1196/annals.1440.011 18400922

[B10] Leech R Kamourieh S Beckmann CF Sharp DJ Fractionating the default mode network: distinct contributions of the ventral and dorsal posterior cingulate cortex to cognitive control J Neurosci 2011 31 3217 3224 10.1523/JNEUROSCI.5626-10.2011 21368033 PMC6623935

[B11] Hinds O Thompson TW Ghosh S Yoo JJ Whitfield-Gabrieli S Triantafyllou C Gabrieli JDE Roles of default-mode network and supplementary motor area in human vigilance performance: evidence from real-time fMRI J Neurophysiol 2013 109 1250 1258 10.1152/jn.00533.2011 23236006

[B12] Bonnelle V Leech R Kinnunen KM Ham TE Beckmann CF Boissezon XD Greenwood RJ Sharp DJ Default mode network connectivity predicts sustained attention deficits after traumatic brain injury J Neurosci 2011 31 13442 13451 10.1523/JNEUROSCI.1163-11.2011 21940437 PMC6623308

[B13] Broyd SJ Demanuele C Debener S Helps SK James CJ Sonuga-Barke EJS Default-mode brain dysfunction in mental disorders: a systematic review Neurosci Biobehav Rev 2009 33 279 296 10.1016/j.neubiorev.2008.09.002 18824195

[B14] Bell SM McCallum RS Cox EA Toward a research-based assessment of dyslexia: using cognitive measures to identify reading disabilities J Learn Disabil 2003 36 505 516 10.1177/00222194030360060201 15493433

[B15] Riccio CA Cohen MJ Garrison T Smith B Auditory processing measures: correlation with neuropsychological measures of attention, memory, and behavior Child Neuropsychol 2005 11 363 372 10.1080/09297040490916956 16051564

[B16] Chermak GD Somers EK Seikel JA Behavioral signs of central auditory processing disorder and attention deficit hyperactivity disorder J Am Acad Audiol 1998 9 78 84 9493945

[B17] Hoeksma MR Kenemans JL Kemner C van Engeland H Variability in spatial normalization of pediatric and adult brain images Clin Neurophysiol 2005 116 1188 1194 10.1016/j.clinph.2004.12.021 15826861

[B18] Castellanos FX Margulies DS Kelly AMC Uddin LQ Ghaffari M Kirsch A Shaw D Shehzad Z Di Martino A Biswal B Sonuga-Barke EJS Rotrosen J Adler LA Milham MP Cingulate - precuneus interactions: a new locus of dysfunction in adult attention-deficit/hyperactivity disorder Biol Psychiatry 2008 63 332 337 10.1016/j.biopsych.2007.06.025 17888409 PMC2745053

[B19] Cao Q Zang Y Sun L Sui M Long X Zou Q Wang Y Abnormal neural activity in children with attention deficit hyperactivity disorder: a resting-state functional magnetic resonance imaging study Neuroreport 2006 17 1033 1036 10.1097/01.wnr.0000224769.92454.5d 16791098

[B20] Weissman DH Roberts KC Visscher KM Woldorff MG The neural bases of momentary lapses in attention Nat Neurosci 2006 9 971 978 10.1038/nn1727 16767087

[B21] Sonuga-Barke EJS Castellanos FX Spontaneous attentional fluctuations in impaired states and pathological conditions: a neurobiological hypothesis Neurosci Biobehav Rev 2007 31 977 986 10.1016/j.neubiorev.2007.02.005 17445893

[B22] Calhoun VD Adali T Pearlson GD Pekar JJ A method for making group inferences from functional MRI data using independent component analysis Hum Brain Mapp 2001 14 140 151 10.1002/hbm.1048 11559959 PMC6871952

[B23] Ding X Lee S-W Cocaine addiction related reproducible brain regions of abnormal default-mode network functional connectivity: a group ICA study with different model orders Neurosci Lett 2013 548 110 114 23707901 10.1016/j.neulet.2013.05.029

[B24] Allen EA Damaraju E Plis SM Erhardt EB Eichele T Calhoun VD Tracking whole-brain connectivity dynamics in the resting state Cereb Cortex 2014 24 663 676 10.1093/cercor/bhs352 23146964 PMC3920766

[B25] Damoiseaux JS Beckmann CF Arigita EJ Barkhof F Scheltens P Stam CJ Smith SM Rombouts SA Reduced resting-state brain activity in the “default network” in normal aging Cereb Cortex 2008 18 1856 1864 10.1093/cercor/bhm207 18063564

[B26] Raichle ME Snyder AZ A default mode of brain function: a brief history of an evolving idea NeuroImage 2007 37 1083 1090 10.1016/j.neuroimage.2007.02.041 17719799

[B27] Lancaster JL Woldorff MG Parsons LM Liotti M Freitas CS Rainey L Kochunov PV Nickerson D Mikiten SA Fox PT Automated Talairach atlas labels for functional brain mapping Hum Brain Mapp 2000 10 120 131 10.1002/1097-0193(200007)10:3<120::AID-HBM30>3.0.CO;2-8 10912591 PMC6871915

[B28] Van Dijk KRA Sabuncu MR Buckner RL The influence of head motion on intrinsic functional connectivity MRI NeuroImage 2012 59 431 438 10.1016/j.neuroimage.2011.07.044 21810475 PMC3683830

[B29] Power JD Mitra A Laumann TO Snyder AZ Schlaggar BL Petersen SE Methods to detect, characterize, and remove motion artifact in resting state fMRI NeuroImage 2014 84 320 341 23994314 10.1016/j.neuroimage.2013.08.048 PMC3849338

[B30] Rombouts SARB Damoiseaux JS Goekoop R Barkhof F Scheltens P Smith SM Beckmann CF Model‒free group analysis shows altered BOLD FMRI networks in dementia Hum Brain Mapp 2009 30 256 266 10.1002/hbm.20505 18041738 PMC6870626

[B31] Leech R Braga R Sharp DJ Echoes of the brain within the posterior cingulate cortex J Neurosci Off J Soc Neurosci 2012 32 215 222 10.1523/JNEUROSCI.3689-11.2012 PMC6621313 22219283

[B32] Pearson JM Heilbronner SR Barack DL Hayden BY Platt ML Posterior cingulate cortex: adapting behavior to a changing world Trends Cogn Sci 2011 15 143 151 10.1016/j.tics.2011.02.002 21420893 PMC3070780

[B33] Uddin LQ Kelly AMC Biswal BB Margulies DS Shehzad Z Shaw D Ghaffari M Rotrosen J Adler LA Castellanos FX Milham MP Network homogeneity reveals decreased integrity of default-mode network in ADHD J Neurosci Methods 2008 169 249 254 10.1016/j.jneumeth.2007.11.031 18190970

[B34] Cacace AT McFarland DJ The importance of modality specificity in diagnosing central auditory processing disorder Am J Audiol 2005 14 112 123 10.1044/1059-0889(2005/012) 16489868

[B35] Power JD Barnes KA Snyder AZ Schlaggar BL Petersen SE Spurious but systematic correlations in functional connectivity MRI networks arise from subject motion Neuroimage 2012 59 2142 2154 10.1016/j.neuroimage.2011.10.018 22019881 PMC3254728

[B36] De Celis Alonso B Hidalgo Tobón S Dies Suarez P García Flores J De Celis Carrillo B Barragán Pérez E A multi-methodological MR resting state network analysis to assess the changes in brain physiology of children with ADHD PLoS One 2014 9 e99119 10.1371/journal.pone.0099119 24945408 PMC4063721

[B37] Cole DM Smith SM Beckmann CF Advances and pitfalls in the analysis and interpretation of resting-state FMRI data Front Syst Neurosci 2010 4 8 20407579 10.3389/fnsys.2010.00008 PMC2854531

[B38] Beckmann CF Smith SM Tensorial extensions of independent component analysis for multisubject FMRI analysis NeuroImage 2005 25 294 311 10.1016/j.neuroimage.2004.10.043 15734364

[B39] Liu C-H Ma X Li F Wang Y-J Tie C-L Li S-F Chen T-L Fan T Zhang Y Dong J Yao L Wu X Wang C-Y Regional homogeneity within the default mode network in bipolar depression: a resting-state functional magnetic resonance imaging study PLoS One 2012 7 e48181 10.1371/journal.pone.0048181 23133615 PMC3487908

[B40] Von dem Hagen EAH Stoyanova RS Baron-Cohen S Calder AJ Reduced functional connectivity within and between “social” resting state networks in autism spectrum conditions Soc Cogn Affect Neurosci 2013 8 6 694 701 10.1093/scan/nss053 22563003 PMC3739917

[B41] Valsasina P Rocca M Misci P Pagani E Falini A Filippi M Proceedings of The International Society for Magnetic Resonance in Medicine 2009 Honolulu, Hawai’i, USA

